# Haemodynamic forces in aortic stenosis between myocardial function and valvular obstruction

**DOI:** 10.1093/ehjimp/qyag175

**Published:** 2026-10-09

**Authors:** Giovanni Tonti, Eduardo Bossone, Gianni Pedrizzetti

**Affiliations:** Department of Cardiovascular Disease, University G. D’Annunzio, Chieti–Pescara, Italy; Heart Centre of Excellence, King Faisal Specialist Hospital & Research Center, Riyadh, Saudi Arabia; Department of Public Health, University of Naples ‘Federico II’, Naples, Italy; Department of Engineering and Architecture, University of Trieste, Trieste, Italy

**This editorial refers to ‘Feasibility and clinical significance of cardiovascular magnetic resonance derived hemodynamic force assessment in aortic valve stenosis’, by S.J. Backhaus *et al.***

The study by Backhaus *et al.*^1^ enters a central debate in aortic stenosis: the search for the optimal timing of valve replacement has shifted attention from the valve to the myocardium. Symptoms and left ventricular ejection fraction (LVEF) remain decisive, but both may identify decompensation only after prolonged pressure overload has altered ventricular structure, energetics, and reserve. Strain, biomarkers, and cardiovascular magnetic resonance (CMR) tissue characterization have therefore been proposed as earlier markers of injury.^2^ Haemodynamic forces (HDF) offer a different perspective. They estimate the magnitude and direction of forces exchanged between moving blood and the ventricular boundaries. Since the calculation of HDF from regular Cine CMR requires the delineation of the orifices crossed by flow, Backhaus *et al.* ask a deceptively simple question: which outflow boundary should be used when HDF are calculated in a ventricle ejecting through a stenotic valve?

Image-based HDF are derived from the balance of momentum within a defined ventricular volume.^3,4^ Endocardial motion supplies the transformation of geometric changes into blood momentum, while mitral and outflow areas account for momentum crossing its boundaries. The conventional systolic outlet is the left ventricular outflow tract (LVOT). Backhaus *et al.* tested the difference when replacing it with the smaller aortic valve area (AVA) measured by computed tomography in patients undergoing transcatheter aortic valve implantation (TAVI). This boundary change markedly increases systolic impulse, whereas the systolic–diastolic transition and diastolic components change little. In patients with preserved LVEF, LVOT-derived values resemble those of controls, whereas AVA-derived forces are substantially higher. Yet AVA correction leaves the extracellular volume calculation (ECV) and six-minute walking-distance associations unchanged and shifts short-term outcome prediction only from trend to significance without changing long-term prognosis. This dissociation is the central observation.

The result is physiologically meaningful. HDF represent the volume-averaged intraventricular pressure gradient. Between the LVOT and the stenotic orifice, pressure is converted into kinetic energy, and a steep systolic gradient develops. The AVA formulation is therefore not simply a corrected LVOT formulation. It combines an intracavitary component, which contains information on ventricular function, with an added transvalvular component that predominantly reflects the valve (*Figure 1A*). The two estimates retain similar clinical associations because they share the same myocardial component; the valvular term adds a contribution in systole, proportional to the degree of stenosis, but brings little prognostic information in this cohort (*Figure 1B*). For assessing ventricular function, the conventional LVOT boundary remains the more internally consistent formulation as momentum is balanced within a volume bounded basally by the LVOT, not the valve, whose area lies outside the segmented boundary.

**Figure 1:**
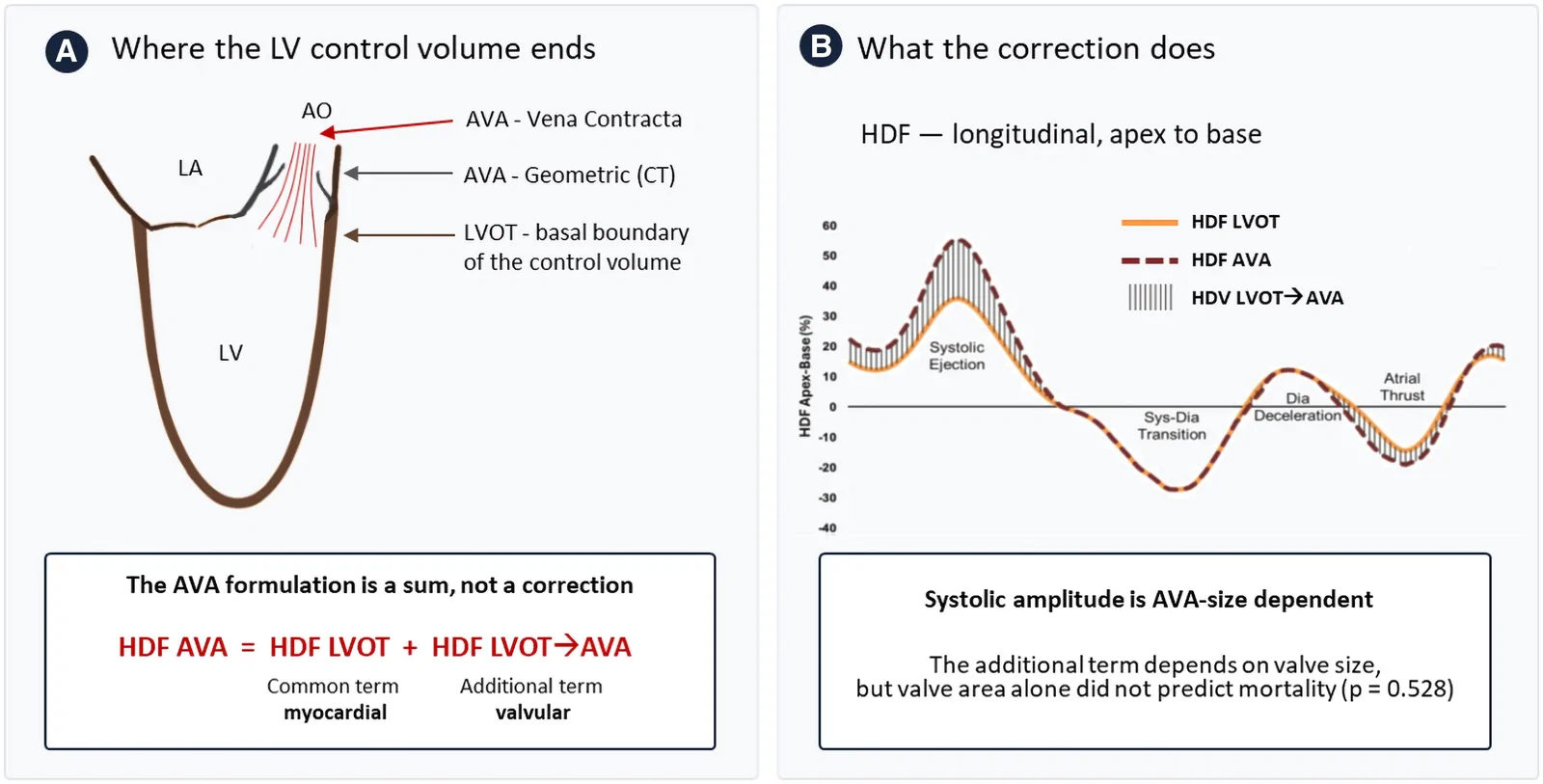
(*A*) The LV haemodynamic force (HDF LVOT) is computed inside the left ventricle (LV) bounded at the base by the LVOT. Between the LVOT and the stenotic orifice, pressure is converted into kinetic energy, and a steep systolic gradient develops from the LVOT to the aortic valve area (AVA); this contribution (HDF LVOT→AVA) is added to the previous contribution to give the HDF AVA. (*B*) The two HDF LVOT and HDF AVA share the same myocardial component, and the valvular term adds a systolic contribution to the latter, proportional to the degree of stenosis, bringing little incremental information other than the valve size.

A further ambiguity may affect the identification of the AVA, in general.^5^ In the present study, baseline echocardiography provides an effective orifice area at the vena contracta, whereas the area entered into the computation is a computed tomography-derived geometric area (*Figure 1A*). Their ratio is the contraction coefficient and varies with leaflet geometry, calcific distortion, inflow shape, and flow. The same geometric area may therefore generate different effective areas and different momentum fluxes.

Despite this, the study has important strengths: prospective recruitment, integrated CMR and computed tomography, blinded post-processing, and extended follow-up. The direct comparison of LVOT- and AVA-derived HDF tests an assumption usually left implicit. The preservation of clinical associations despite a large change in scale suggests that patient ranking is driven mainly by ventricular motion and geometry rather than by the selected outlet area.

The prognostic findings require greater caution. Events were limited, and the models remained univariable. The association between systolic force and mortality should therefore remain hypothesis generating rather than be read as evidence that a systolic HDF metric independently identifies treatment timing. Two further confounders deserve attention: force amplitude scales with flow, so lower systolic HDF may partly restate the prognosis of low flow rather than a separate mechanical signal; and concomitant aortic or mitral regurgitation can perturb the assumed boundaries, particularly the inflow boundary from which atrial thrust, the principal long-term HDF signal, is derived. On this aspect, a further informative result may arise outside ventricular systole. Atrial thrust showed the strongest associations with ECV, walking distance, and longer-term mortality, irrespective of the outflow area used. This is physiologically coherent: changing the systolic outlet does not alter the mitral boundary. Atrial contraction may initially compensate for impaired early filling in a stiff ventricle; subsequent attenuation may signal exhausted reserve, atrial myopathy, or advanced ventricular–atrial coupling abnormalities.

Two issues must be resolved before this finding can be translated. The first is definitional: the convention used to delimit late diastole determines the value of the integral and is essential for reproducibility. The second is incremental value. Reduced early filling supported by augmented atrial transport is already captured, at least partly, by transmitral E, A, and E/A or by an equivalent partition of the volume–rate curve. Whether atrial thrust adds information beyond these simpler indices cannot be established without prospectively aligned diastolic assessment.

Beyond this, the most useful implication of this work may be the identification of descriptors that are less sensitive to the boundary problem. Because the transvalvular term scales with instantaneous flow, it is confined largely to ejection and vanishes as flow ceases. It inflates systolic amplitude while leaving the zero crossings, and therefore the principal temporal landmarks, relatively unchanged. Amplitude is boundary dependent; timing, duration, and vector morphology may be more robust. This observation suggests shifting the attention from how large the force is to when, and for how long, it is generated. Relating force timing to its amplitude may additionally distinguish a narrow, spiked waveform from a broader, plateau-like profile.

The systolic–diastolic transition should also be disaggregated. Its negative excursion may combine terminal ejection deceleration, the base-to-apex force associated with relaxation, suction, and the acceleration of rapid filling. Their sum may remain unchanged even when their components move in opposite directions, as might occur in a hypertrophied, fibrotic ventricle. The same post-processing may provide the required landmarks without additional image acquisition, but this needs explicit methodological standardization.

The proposed trajectory follows the natural history of pressure overload. During early compensation, rising valvular load may be met by brisk or augmented longitudinal force while LVEF remains preserved. As hypertrophy, altered geometry, microvascular dysfunction, and interstitial expansion develop, force generation may become slower and ejection more prolonged. Peak force may fall and time to peak may lengthen, while a broader waveform initially preserves total impulse. With advanced fibrosis and declining reserve, impulse and atrial thrust may also fall. Peak amplitude alone could therefore change non-monotonically; the within-patient temporal pattern may be more informative.

Clinically, the biological case for early sensitivity is plausible: longitudinal forces decline across mild, moderate, and severe stenosis and predict valve replacement or death despite preserved LVEF.^6^ Yet sensitivity to loading is also a limitation, because rapid changes may reflect reversible obstruction rather than irreversible myocardial injury. Early TAVI favoured intervention over surveillance in asymptomatic severe stenosis,^7^ whereas later implants did not significantly improve primary outcomes when intervention was guided by fibrosis.^8^ Prognostic enrichment does not necessarily identify treatment benefit, and current guidelines appropriately retain an integrated approach.^9^

This cohort cannot establish HDF-guided timing: patients had already been referred for TAVI, so the clinical threshold had largely been crossed, although systolic function remained relatively preserved. A single post-decision measurement cannot show whether HDF change precedes fixed myocardial injury; their value may instead lie in serial trajectories. A reproducible change in time to peak, impulse, phase duration, vector orientation, or regional dispersion, aligned with evolving geometry, ECV, and symptoms, could mark the transition from adaptive hypertrophy to early maladaptation. Because within-patient follow-up fixes the boundary convention, it may also sidestep much of the methodological ambiguity raised here (*Table 1*).

**Table 1:** Evidence required before haemodynamic forces (HDF) can guide intervention timing

Requirement	Clinical question	Current evidence
Construct validity	Does the metric separate ventricular adaptation from valve obstruction?	Systolic amplitude depends strongly on the selected outlet boundary.
Boundary specification	Which orifice should define the systolic boundary?	A planimetric geometric area is used; comparison with effective orifice area or energy-loss coefficient remains unresolved.
Vector and phase definition	Do timing, orientation, and dispersion descriptors survive a change of formulation?	Time to peak, phase duration, vector angle, transverse components, temporal dispersion, and regional forces are not analysed.
Confounding	Are flow state, regurgitation, and comorbidity accounted for?	Flow dependence and concomitant regurgitation may affect interpretation; models remain insufficiently adjusted.
Incremental value	Does atrial force add to diastolic Doppler or volume–rate indices?	Not testable without prospectively aligned diastolic assessment.
Treatment interaction	Do serial HDF changes predict benefit from early replacement?	One pre-intervention time point; no HDF-guided trial.

In conclusion, Backhaus *et al.* show that the choice of outflow boundary profoundly changes the magnitude of systolic HDF but not their main clinical associations. The finding is reassuring for measurement and cautionary for interpretation: the added term is predominantly valvular, whereas the shared term is myocardial. HDF may ultimately identify when the pressure-loaded ventricle ceases to compensate, but amplitude alone is unlikely to suffice. The more promising signal is a serial change in timing, duration, impulse, orientation, transverse dispersion, and regional force organization that precedes fixed fibrosis or loss of conventional function. Defining where the ventricle ends remains essential to understanding what the force means, and whether it can tell us when to intervene.

## Data Availability

The data underlying this article will be shared on reasonable request to the corresponding author.
